# How to Assess Drugs in the Treatment of Acute Bipolar Mania?

**DOI:** 10.3389/fphar.2013.00004

**Published:** 2013-01-29

**Authors:** Michel Bourin, Florence Thibaut

**Affiliations:** ^1^Neurobiologie de l’Anxiété et de la Dépression, Faculté de Médecine, Université de NantesNantes, France; ^2^Department of Psychiatry, Faculté de Médecine, CHU de Rouen, INSERM U 614Rouen, France

**Keywords:** bipolar disorder, mania, evaluation criteria, anticonvulsant, atypical antipsychotics, mood stabilizers

## Abstract

Bipolar affective disorder is a serious mental disease associated with significant morbidity and mortality. Good-quality research available to guide treatment strategies remains insufficient, particularly with regard to manic or hypomanic episodes. A critical review of the various stages of mania might be helpful for pharmaceutical companies and investigators as a prerequisite for the clinical evaluation of potential antimanic properties of medications. The main difficulty is with a comparison between anticonvulsants, antipsychotics, and mood stabilizers such as lithium (with equal efficacy in the acute phase and the prevention of recurrent manic episodes). No consensus has been reached with regard to the treatment of bouts of acute mania in various parts of the world. Controlled clinical trials have, at last, provided irrefutable evidence of the activity of lithium, which has long been used alone, as well as that of divalproate or its derivatives and, to a lesser extent, carbamazepine. The new antipsychotic agents have more recently established their efficacy, especially aripiprazole, asenapine, quetiapine; olanzapine, risperidone, and ziprasidone (not sure where the paradox is). In Europe, haloperidol is still the reference substance used in clinical trials despite the fact that it is not officially indicated in the treatment of mania. In the USA, lithium, divalproate, or antipsychotics can be prescribed as first-line treatment. In Europe, lithium remains the first-line medication, whereas divalproate and atypical antipsychotic agents are used only as second-line therapy. Although both types of medication (antipsychotics, normothymic agents, and/or anticonvulsants) have proved to be clinically effective in the management of mania by reducing the mania scores overall, the same does not apply, however, to all symptoms of mania. Factorial approaches to mania have all shown that since there are several clinical forms of mania, several clusters of manic symptoms can be identified. Antipsychotic and normothymic agents and/or anticonvulsants do not appear to have the same effects on each of these identifiable clusters of symptoms, mainly psychotic features. We believe that it is vitally important for future clinical trials of mania treatment to focus on the treatment effect by adopting a factorial approach to characterization of the episode using an appropriate methodological structure. These questions highlight the uncertainty shrouding the very structure of manic episodes, namely that these are predominantly of a thymic or psychotic nature. The Europeans undoubtedly consider mania to be more of a thymic episode and prefer lithium as the first-line treatment, whereas the Americans believe that psychotic symptoms dominate and widely prescribe antipsychotic agents. However, from the standpoint of clinical trials currently available, even though antipsychotic agents are certainly effective in reducing the scores on the mania scales, it is not clear whether they can be considered purely as antimania treatments.

## Introduction

Mania and depression have been considered as distinct, yet related, phenomena since antiquity (Angst and Marneros, 2001). Only in recent history, mood disorders have been divided into two distinct syndromes: mania and depression. As the father of current psychiatric nosology, Kraepelin was one of the first to subtype manic subjects into those with and without depression. From Kraepelin, until the second version of the Diagnostic and Statistical Manual of Mental Disorders (DSM-II; American Psychiatric Association, 1968), both syndromes were considered as mood disorders including different subtypes such as recurrent mania, recurrent depression, recurrent mania, and depression, and affective disorders with mixed states. Later on, DSM-III made a distinction between major depressive disorder and bipolar disorder defined by the presence of mania. Several reasons have led to consider bipolar disorder and unipolar disorder as distinct illnesses. Furthermore, increasing evidence has supported a distinct etiology and a different lifetime course for mania (more severe) as compared to depression.

Mania and depression are considered as opposite poles of a continuum with grandiose self-esteem and self-perceived abilities on one side, and feelings of self-loathing, incompetence, and apathy on the other. These two opposite states of mood can be observed at different periods of time in the same bipolar patient.

Bipolar patients seem to experience greater mood reactivity to environmental circumstances as compared to controls. This hyper reactivity may be more pronounced for positive stimuli. Indeed, during manic episodes, bipolar subjects remember the positive moments of their life much more readily than the negative ones, and they perceive themselves much more positively than they would at other times. They show large increases in self-confidence after initial successes or when they are experiencing hypomanic symptoms. After small successes or in the presence of mild hypomanic symptoms, subjects with a bipolar history or a vulnerability to the disease appear to perceive their life goals as more attainable, they are more likely to tackle difficult tasks, and they are less likely to perceive danger. Interestingly, these processes appear to require a trigger: bipolar subjects do not appear to exhibit robustly elevated self-confidence in their success during euthymic periods in the absence of recent success or positive moods. Hence, success seems to promote greater goal pursuit among persons vulnerable to bipolar disorder. This may contribute to explain the higher risk of manic symptoms after major life successes, described above (Johnson et al., 2000). It appears also that elevated goal pursuit predicts increase in manic episodes recurrence over time (Lozano and Johnson, 2001). Even though bipolar subjects appear to endorse higher than average levels of goal setting, and higher self-confidence during mania, one might expect the distribution of their scores to overlap with the general population. Is the association of permanent high goals, liability of emotional reactions, and increased self-confidence after success sufficient to account for the spiral into mania? Or is something else needed? Basic research suggests that dopaminergic pathways from the nucleus accumbens to the prefrontal cortex are activated when subjects anticipate a reward (Knutson et al., 2001). Bipolar disorder has been hypothesized to result from a dysregulation of this pathway (Hestenes, 1992; Depue et al., 1996). Antipsychotic drugs have shown a good efficacy in the treatment of mania. Hence, one might expect that bipolar patients may have greater difficulties in achieving emotional and behavioral control which challenge the reward system.

Since 2005, many guidelines have been published [World Federation of Societies of Biological Psychiatry (Grunze et al., 2009); the Canadian Network for Mood and Anxiety Treatments (CANMAT) and the International Society for Bipolar Disorders (Yatham et al., 2009), the British Association for Psychopharmacology (BAP; Goodwin and Consensus Group of the British Association for Psychopharmacology, 2009), the National Institute for Health and Clinical Excellence (NICE) (2009), and the Clinical Practice Recommendation for bipolar disorder (Malhi et al., 2009)]. Nivoli et al. (2012) have published an interesting critical review of the existing guidelines focusing on the treatment of manic/hypomanic and mixed episodes. They came to the conclusion that there is a widespread inconsistency in diagnostic assessment of different subtypes and definitions with respect to bipolar disease across guidelines and when specific diagnostic criteria are proposed, as in DSM IV and ICD 10, they are insufficiently representative of the clinical complexity of the bipolar disorder patient. In the first part of this paper we will discuss the current diagnostic criteria.

All guidelines agree that the first-line treatment of manic/hypomanic and mixed episodes should be lithium, valproate, or atypical antipsychotics as monotherapy. Combination therapy including lithium or valproate with an atypical antipsychotic is suggested as a second-line choice, and sometimes as first choice treatment in cases of severe mania. In the second part of this paper we will discuss the different mood stabilizers and atypical antipsychotics that might be used in mania in this paper and the limitations of the current research conducted in this field.

## Classical Clinical Descriptions

Manic states are typically characterized by abnormally and persistently elevated, expansive, or irritable mood lasting at least 1 week associated with different combinations of the following symptoms: inflated self-esteem or grandiosity, subjects are more talkative than usual or make pressure to keep talking, they speak faster, they have flight of ideas or subjective experience that thoughts are racing, quicker thought, higher distractibility, psychomotor agitation, higher energy (with a corresponding decreased need for sleep), irritability, increased perceptions, excessive involvement in pleasurable activities with a high potential of harmful consequences (e.g., engaging in unrestrained buying sprees, sexual indiscretions, or foolish business investments). The degree, type, and chronicity of these cognitive, perceptual, and behavioral changes determine the subtype of mania, namely hypomania, or mania.

In hypomania, the above changes are generally moderate and may or may not result in serious problems for the individual experiencing them. In more severe episodes, however, they profoundly disrupt the lives of the patients, their families, and the society.

In acute mania, mood was not accurately described by the classical authors, perhaps because extreme changes in cognition and behavior are more easily observable than are subjective mood states. Two thousand years ago, however, Aretaeus of Cappadocia noted that those who were manic were active, and expansive. They were naturally joyous, they laughed, and they joked: “they show off in public with crowned heads as if they were returning victorious from the games; sometimes they laugh and dance all day and night.” Few centuries later, Kraepelin (1921) and more recently, Roccatagliata (1986) agreed, but have stressed the unstable presentation of manic mood: “Mood is unrestrained, merry, exultant, occasionally visionary of pompous, but always subject to frequent variation, easily changing to irritability and irascibility or even to lamentation and weeping.” Activity and behavior are greatly increased and diversified in mania. Patients are tireless, rash, virulently opinionated, and may show aggressiveness. For many patients, excessive energy translates directly into pressured writing and an inordinate production of written declaration, poetry, and artwork. Particularly dramatic and extreme among the clinical features of acute mania are the frenetic, seemingly aimless, and occasionally violent behaviors of manic patients. In the same way, strange, impulsive, and grossly inappropriate behavior patterns are frequently observed.

## Diagnosis of Acute Bipolar Mania

The history of psychiatric diagnosis has been notable for its confusion, reflected in the myriad overlapping systems used for classifying and subtyping depressive disorders. However, Kraepelin clarified the diagnosis of depression by gathering all recurrent affective disorders into manic-depressive illness, a broad category later divided into unipolar and bipolar subgroups.

When making the diagnosis, the BAP guidelines suggest the identification of core symptoms of mania as well as the importance of second sources for collateral information (family members, caregivers, etc.). The clinician will combine them together with the patient’s history and previous response to treatment, as well as the family history. Individual symptoms and even clusters of symptoms, examined at one specific time often lack diagnostic specificity, although such cross-sectional views are sometimes the only ones available.

The structure of mania has been examined in recent phenomenological studies using factor analytic and other methods. These studies have revealed that the most common symptoms of mania are motor activation, flight of ideas, pressured speech, and decreased sleep, while elevated mood and increased sexuality are less common. These authors have also identified four types of mania that correspond to Kraepelin’s observations: hypomania, acute mania, delusional mania, and depressive or anxious mania.

## DSM IV Definition of a Manic Episode

Criterion A: a manic episode is a distinct period during which there is an abnormally and persistently elevated, expansive, or irritable mood. This period of abnormal mood must last at least 1 week (or less if the hospitalization is required).

Criterion B: the mood disturbance must be accompanied by at least three additional symptoms from a list that includes inflated self-esteem or grandiosity, decreased need for sleep, pressure of speech, flight of ideas, distractibility, increased involvement in goal-directed activity or psychomotor agitation, and excessive involvement in pleasurable activities with a high potential for painful consequences.

Criterion C: the symptoms do not meet criteria for a mixed episode.

Criterion D: the disturbance must be sufficiently severe to cause marked impairment in social or occupational functioning or to require hospitalization, or it is characterized by presence of psychotic features.

A manic episode can occur during different subtypes of bipolar disorder:

– In type I, the patient presents manic episodes alternating with major depressive episodes (Ahearn and Caroll, 1996).
– In type II, the patient presents recurrent major depressive episodes and at least one or several hypomanic episodes (Hantouche et al., 1998).
– In type III, the patient presents some recurrent major depressive episodes with one or several pharmacologically induced hypomanic episodes.

A particular subtype of manic episode is mania with delirium or psychotic mania, where clinical symptoms of mania are associated with mood-congruent psychotic features. Grandiose delusions may be an expression of severity of mania whereas other delusions might confuse the distinction from schizophrenia and rather correspond to a distinct subtype of mania.

In mixed episode, criteria for a manic episode and a major depressive episode (except for the duration) are fulfilled simultaneously, nearly every day during at least 1 week (Cassidy et al., 2000). The mood disturbance is sufficiently severe to cause marked impairment in occupational functioning, social activities or relationships with others, or to require hospitalization in order to prevent harm to self or others, or the patient has some psychotic features.

Dysphoric mania describes mania with some depressed and dysphoric features that are either not severe enough or insufficiently lasting to fulfill the criteria for a major depressive episode. Concerning treatment, the amount of evidence is rather limited in psychotic mania, dysphoric mania, or mixed episodes.

## Inclusion Criteria for Acute Bipolar Mania

*A priori*, only bipolar I patients may be included in clinical trials. However, the inclusion of hypomanic subjects fulfilling type II or III bipolar disorder criteria might be considered. We could also include only patients with a first manic episode, but they are less numerous, and might be sometimes difficult to differentiate from schizo-affective patients. Schizo-affective patients might require the use of two different scales for diagnosis and assessment SADS-C (Endicott and Spitzer, 1978) and Young-Mania-Rating-Scale (YMRS; Young et al., 1978).

Is it necessary to include patients presenting psychiatric co-morbidities in clinical trials? Comorbidity is frequently observed in bipolar patients:

– 17–35% Have a generalized anxiety disorder (GAD),
– 19% Have panic attacks,
– 9% Have obsessive compulsive disorder (OCD),
– 20% Have social anxiety disorder.

Do these co-morbidities influence the therapeutic response to a given medication? In clinical practice, it is difficult to either include or exclude these patients where the investigator does not necessarily know the past psychiatric history. The use of the MINI could help to distinguish between a past history of a comorbid disorder and current comorbid symptoms associated with the manic episode. However, the MINI (Sheehan et al., 1998) is not suitable for the assessment of comorbid psychotic features.

## What is the Minimal Treatment Duration Required for a Clinical Trial during an Acute Manic Episode?

In most studies, the duration of the trial is 4 weeks, and the patients are either hospitalized or not. Although manic episodes improve in general quickly enough, this duration does not allow distinguishing a simple remission from cycling patients. Rapid cyclers (>4 cycles of depression or mania per year) have a particularly labile mood. It is crucial to study the efficacy of a specific treatment during the acute phase but also to identify potential switches from manic episode to depression. In order to answer this question, duration of clinical trials of 6 weeks rather than 4 may be considered.

This 6-week-period should be followed by a maintenance phase for another 6 weeks in order to include potential relapses. It seems therefore necessary to plan an overall length of 12 weeks (6 weeks for the acute phase and 6 weeks for the maintenance phase).

In addition, long term studies are also necessary to provide scientific data about the minimal duration of treatment required after a manic episode (Yatham et al., 2012).

## Exclusion Criteria for Acute Bipolar Mania

In controlled studies, comorbid, suicidal, medically ill patients are excluded, however, the data are lacking concerning the management of these patients by psychiatrists.

The exclusion criteria should be as follows:

– Suicidal risk.
– First manic episode.
– Bipolar I, rapid cycling (more than 4 cycles per year).
– Patients whose manic episode started more than 4 weeks earlier and patients with psychotic symptoms, or a manic episode caused by substances, medications, or a general medical condition.
– Manic symptoms caused by psychotropic drugs including benzodiazepines used during the previous 3 months.
– Past history of delirium, schizophrenia, schizo-affective episodes, or personality disorders according to DSM IV criteria.

The adjunctive treatments will have to be adequately reported. It seems reasonable to exclude patients who were receiving long half-life antidepressant treatment before inclusion such as fluoxetine or even sertraline and patients for whom plasma concentrations of lithium, carbamazepine, or valproate were equal or superior to the efficient well-known concentrations.

The adjunctive use of benzodiazepines, which have no activity on mood, such as lorazepam and oxazepam may be necessary for example when the duration of sleep is lower than 3–4 h.

## Which Assessment Scales Can be Used?

The YMRS is the gold standard for the evaluation of acute manic symptoms during clinical trials.

The minimal score required for inclusion is equal or superior to 20, however, such a score may allow inclusion of patients presenting with hypomania which might introduce a bias. In order to firmly exclude hypomanic patients, a minimum inclusion score equal or superior to 24 seems more appropriate.

Clinical studies usually use a 50% reduction of the YMRS and Mania rating scale (MRS) The MRS can be divided into Mania Symptoms Scale (MSS) and Barratt Impulsiveness Scale (BIS). The MSS allows the assessment of the core symptoms of mania (sleep disorders, increased energy or motor activity, megalomania, increase of finalized activities), and of the elevated mood which is not considered as a cardinal feature of mania. The MSS score required for inclusion should be equal or superior to 15. In case of severe mania, it will be necessary to keep a score equal or superior to 20, with a score superior to 5 on at least two items of this scale.

The additional use of a nurse scale would not increase the strength of the assessment of manic symptoms.

The YMRS will be considered as the main assessment scale in order to evaluate treatment efficacy. Clinical responders will be defined as patients whose improvement on YMRS and MSS as compared to baseline will be at least 50% with a score on the Clinical Global Impression for Bipolar Disorder scale (CGI-BP) mania lower than 4.

The Montgomery–Åsberg Depression Rating Scale (MADRS; Montgomery and Asberg, 1979) or Hamilton Depression Rating Scale (HAMA-D) should be used systematically in order to assess the possible switch toward depression.

Patients with mixed episodes could be included, if they present a YMRS score superior to 24.

## Which Drugs are of Interest?

In this second part of the review the current literature focusing on medications used for the treatment of acute mania is critically reviewed.

### Lithium

Numerous controlled studies have clearly established the superiority of lithium as compared to placebo in acute mania. As a consequence, since the 1990s, studies designed to evaluate newer medications have used lithium as the gold standard treatment (Bowden et al., 1994). A slower onset of action of lithium as compared to other medications has been discussed (Bowden et al., 1994). Most of the placebo-controlled studies varied as regard to dosage, serum levels, and rapidity of dosage titration of lithium, making it difficult to establish the precise time to onset of lithium antimanic action. Nonetheless, there is little doubt that lithium is as effective as other antimanic agents over a 3-week period, while some studies have even reported a quicker onset of action (10 days).

Actually, the largest randomized double-blind study of lithium versus placebo in acutely manic patients was designed to study the benefits of divalproex (valproate) in the treatment of acute mania, with lithium and placebo-treated groups serving as controls (Bowden et al., 1994; Swann et al., 1997, 2000). In this study, nearly 50% of respectively the lithium-treated patients and the valproate-treated group were improved (i.e., >50% reduction of YMRS scores) during the 3-week trial period as compared with about 25% in the placebo group. Nearly half of the patients had a previous history of poor response to lithium, which predicted the differential response observed in this trial. Thus, among the prior lithium responders who were receiving lithium in this trial, there was a 15-point mean reduction in YMRS scores (60% improvement), compared with only a one-point mean improvement in the previously lithium non-responders (Bowden et al., 1994). Among the prior lithium responders who were randomized to valproate, by contrast, there was only a 27% improvement, compared with the 60% improvement observed with lithium.

A meta-analysis of six randomized controlled trials using lithium for the treatment of acute mania reported an overall standardized effect size of 0.40 (95% CI: 0.28–0.53) and an overall numbers-needed-to treat patients for response of 6 (95% CI: 4–13; Storosum et al., 2007).

Plasma levels for lithium in recent controlled studies were usually in the range between 0.6 and 1.3 mmol/l.

### Anticonvulsants

The use of anticonvulsant agents for the treatment of bipolar disorders was a watershed event in psychiatry in the late twentieth century. The effectiveness of some anticonvulsants in patients who did not respond adequately or who could not tolerate lithium has made these drugs a welcome addition to the armamentarium. Controlled trials have shown that both valproate and carbamazepine were more effective than placebo, and as noted above, as effective as lithium for the treatment of acute mania (Post et al., 1986; Small et al., 1991; Bowden et al., 1994, 2006; Weisler et al., 2004, 2005).

In contrast, placebo-controlled data are negative concerning the acute manic phase for gabapentin and topiramate (Fountoulakis et al., 2012).

#### Carbamazepine

Starting in the 1970s, a number of studies have shown that carbamazepine was superior to placebo, although not necessarily equivalent to lithium in the short term treatment of mania. Okuma et al. (1990) have conducted a double-blind placebo-controlled study including manic patients who were receiving antipsychotic treatment without substantial benefit. In half of the 105 patients, 200–400 mg/day of carbamazepine was added to haloperidol, and in the other half, lithium was added to haloperidol (mean lithium plasma concentration: 0.46 meq/l). At endpoint, all patients were moderately to markedly improved. Since all patients were receiving a mood stabilizer in combination with haloperidol, it remains unclear how much of the improvement observed was due to the adjunctive mood stabilizers or to haloperidol. Small et al. (1991) conducted a 8-week double-blind comparison study of carbamazepine and lithium in 52 hospitalized acutely manic patients. They have found that both drugs were equally effective in reducing manic symptoms (as assessed by YMRS scores), a conclusion supported by a later meta-analysis (Emilien et al., 1996). The first large randomized placebo-controlled mania study has shown significant superiority of carbamazepine as compared to placebo in the treatment of acute mania (Weisler et al., 2004, 2005). However, some tolerability issues with rapid titration and its interaction potential with numerous psychiatric and non-psychiatric medications limit its use (Grunze et al., 2009).

Conclusive evidence is lacking for oxcarbazepine (Grunze et al., 2009).

#### Valproate

Valproate is a common generic name used for different preparations (valproic acid, sodium valproate, divalproate, divalproex sodium, and valpromide). Only valproic acid reaches and penetrates the blood brain barrier. The French psychiatrist Lambert first reported a possible role of valproate in the treatment of bipolar disorders during the first clinical trials in patients with epilepsy in the 1960s (Lambert et al., 1966). Many years later, Calabrese and Delucchi (1990) reported that this compound appeared to have marked effect in mania. They also noted that most of the patients (63%) showing a good clinical improvement with valproate had failed to improve previously on lithium, carbamazepine, or both.

Randomized, placebo-controlled studies have compared valproate with placebo and with lithium. In the first study (Pope et al., 1991), 17 patients were randomized to divalproex and 19 to placebo. The divalproex-treated patients have shown a median improvement of 54% (YMRS scale), versus only 5% in the placebo group. Similar benefits were observed using the Global Assessment of Social Scale (GAS) and the Brief Psychiatric Rating Scale (BPRS). However, in the divalproex group, only 24% of the patients completed the 3-week study versus 21% in the placebo group.

In the largest placebo-controlled study of acute mania conducted to date, 179 patients with acute mania were receiving divalproex, lithium, or placebo for 21 days (Bowden et al., 1994) and, as previously outlined in Pope’ study, a large number of patients failed to complete this study. The patients had been separated into four different groups based on a factorial approach to mania, using different clinical and nursing evaluation scales, namely groups of patients with depressive or irritable (hostile) or psychotic or conventional (hyperactivity) forms of the disease. The study results show that the depressive forms of the disorder have a poor response to the two treatments assessed whereas the psychotic and conventional responded well to treatment. The irritable forms had a better response to divalproate than to lithium. This study thus revealed a significant difference in divalproate compared with placebo as regards impulsiveness and hostility (unlike lithium) whereas lithium and divalproate were both significantly more effective than placebo in terms of the hyperactive component of mania.

Plasma levels of 75–99 mg/l or 520–690 mmol/l seem to be associated with the best efficacy/tolerability ratio for valproate and caution should be used in women of child-bearing age (Grunze et al., 2009).

#### Lamotrigine

Lamotrigine is an anticonvulsant with sodium channel blocking activity similar to that of carbamazepine and phenytoin. Case reports and open studies have suggested some efficacy for lamotrigine in the treatment of mania, but this result was not confirmed in controlled studies. A small (30 subjects) 4-week randomized controlled trial has compared lamotrigine with lithium in the treatment of hospitalized manic patients (Ichim et al., 2000). Both lithium and lamotrigine groups shared similar reductions of YMRS scores, but the absence of a placebo group renders the results of this study inconclusive. Other controlled studies conducted in manic patients have found no significant differences between lamotrigine and placebo (Frye et al., 2000).

### Antipsychotics

During the past decade the treatment of mania has significantly changed with the introduction of atypical antipsychotics. Among the atypical antipsychotics, olanzapine, and risperidone have been most extensively studied. Nivoli et al. (2011), in their recent meta-analysis, have reported that monotherapy with atypical antipsychotics is recommended in all guidelines as a first-line choice in the treatment of acute mania because of their sedative properties and their short term side-effect profile. Although not yet proved in meta-analysis, several trials have shown that atypical antipsychotics have also a lower risk of switch to depression.

#### Aripiprazole

This atypical antipsychotic has a pharmacodynamic profile that distinguishes it from other antipsychotics by being a partial agonist rather than an antagonist of dopamine D2 receptors. Aripiprazole has proven to be an effective medication for the acute treatment of manic and mixed episodes.

Keck et al. (2003) have conducted a 3-week double-blind, placebo-controlled study using aripiprazole in the treatment of mania in 262 hospitalized patients. They have found a 40% response rate (defined as a decrease of 50% or more of YMRS scores) compared with 19% in the placebo group. These findings were subsequently replicated in a second multicentre 3-week study involving 272 manic patients (135 on aripiprazole, 137 on placebo). On the basis of these results, the FDA has approved aripiprazole for the treatment of acute mania.

Vieta et al. (2005) have conducted a double-blind, controlled study of aripiprazole and haloperidol in patients with bipolar I disorder experiencing acute manic or mixed episodes. Patients (*n* = 347) were randomized to receive aripiprazole or haloperidol in this 12-week, multicentre study. The primary outcome measure was the number of patients in response (greater, similar 50% improvement from baseline in YMRS score) and receiving therapy at week 12. At week 12, significantly more patients taking aripiprazole (49.7%) were in response and receiving therapy compared with those taking haloperidol (28.4%; *P* < 0.001). Continuation rates differed markedly between treatments (week 12: aripiprazole, 50.9%; haloperidol, 29.1%). Extrapyramidal adverse events were more frequent with haloperidol than aripiprazole (62.7 versus 24.0%). Aripiprazole showed superior levels of response and tolerability to haloperidol in the treatment of an acute manic episode for up to 12 weeks.

#### Asenapine

Asenapine is a novel pharmacological agent available in sublingual formulations that binds with high affinity and specificity to numerous dopamine, serotonin, noradrenaline (norepinephrine), and histamine receptor subtypes (Weber and McCormack, 2009).

In two large (*n* = 480), well designed, 3-week trials conducted in adult patients with bipolar I disorder, asenapine used in monotherapy was significantly more effective than placebo in improving manic symptoms as assessed using the YMRS total score (primary endpoint) and the CGI-BP. Significant differences between the asenapine and placebo groups were observed after 2 days of treatment (Chwieduk and Scott, 2011). In one trial, both response and remission rates exceeded those observed in the placebo group. In a 9-week extension study, comparing asenapine and olanzapine, including completers from these monotherapy trials, there were no significant differences between asenapine and olanzapine using the MADRS scores, the CGI-BP mania severity scores, the YMRS response rates, and the YMRS remission rates during the extension phase. In the extension study, the efficacy of asenapine in monotherapy appeared to be maintained during 40 weeks (total treatment duration of 52 weeks). In a 12-week trial using asenapine as an adjunctive therapy to lithium or valproate (Szegedi et al., 2012), asenapine was more effective than placebo in improving manic symptoms, based on the YMRS total score at week 3 (primary endpoint). Most of the adverse events associated with asenapine were of mild to moderate severity, with less than 7% of the patients receiving asenapine experiencing serious adverse events (versus 7% with placebo). In a pooled analysis of the monotherapy trials, the most common adverse events reported during the acute phase of asenapine treatment for bipolar mania were: somnolence, dizziness, extrapyramidal symptoms (EPS; other than akathisia), and increased body weight. These side effects were occurring in less than 5% of the patients which was twice the incidence observed in the placebo group. The same side effects were observed during longer term monotherapy with asenapine without any worsening of the severity of EPSs. Asenapine seems to have minimal effects on plasma glucose or lipid measurements and prolactin levels over both short and longer term treatment periods, and had little pro-arrhythmogenic potential. However, further studies using active comparators as well as longer term tolerability and safety data are required. In the meantime, asenapine is a further option for the management of manic and/or mixed symptoms in patients with bipolar I disorder.

#### Haloperidol

Haloperidol is an old classical antipsychotic agent that remains widely used. In Europe, it was considered as the gold standard treatment for acute mania during decades. In recent and well designed studies, this agent has demonstrated acute antimanic efficacy. Prophylactic utility of this agent has not been adequately examined in long term studies.

There was some evidence that haloperidol was more efficient than placebo in terms of reduction of manic and psychotic symptom scores, when used both as monotherapy and as add-on treatment to lithium or valproate. There was no evidence of any difference in terms of efficacy between haloperidol and risperidone, olanzapine, valproate, carbamazepine, sultopride, or zuclopentixol. There was a statistically significant difference between haloperidol and aripiprazole in favor of the latter. No comparative efficacy data were reported with quetiapine, lithium, or chlorpromazine.

To assess the efficacy of haloperidol in the treatment of mania, Cipriani et al. (2006), have completed a meta-analysis of randomized trials comparing haloperidol with placebo or other active compounds in the treatment of acute manic or mixed episodes in patients with bipolar disorder either as monotherapy or as an add-on therapy. Fifteen trials involving 2022 people were included. Compared to placebo, haloperidol was more effective in reducing manic symptoms, both as monotherapy [Weighted Mean Difference (WMD) 5.85 (95% CI: 4–7.69)] and as adjunctive treatment as compared to lithium or valproate [WMD 5.20 (95% CI: 1.14–9.26)]. Haloperidol was significantly less effective as compared to aripiprazole [RR: 1.45 (95% CI: 1.22–1.73)]. No significant differences were observed between haloperidol, risperidone, olanzapine, carbamazepine, or valproate. Compared with placebo, a statistically significant difference in favor of haloperidol was reported in failure to complete treatment [RR: 0.74 (95% CI: 0.57–0.96)]. Haloperidol was associated with less weight gain than olanzapine [RR: 0.28 (95% CI: 0.12–0.67)], but with a higher incidence of tremor [RR: 3.01 (95% CI: 1.55–5.84)] and other movement disorders. Haloperidol caused more EPS than valproate but no difference was found between haloperidol and lithium, carbamazepine, or risperidone in terms of side effects profile.

The authors concluded that there is some evidence that haloperidol is an effective treatment for acute mania. From the limited data available, there was no difference in overall efficacy of treatment between haloperidol and olanzapine or risperidone. Some evidence suggests that haloperidol could be less effective than aripiprazole. Concerning tolerability, clinicians, and patients should be aware of the differing side effect profiles of these compounds. Typical antipsychotics may be more likely to induce depressive symptoms as compared to atypicals (Tohen et al., 2003). In addition, the use of haloperidol is clearly limited by its propensity to induce acute EPSs and later on tardive dyskinesia. Naturalistic data have suggested that bipolar patients may be at higher risk for these side effects as compared to schizophrenic patients (Keck et al., 2000).

#### Olanzapine

This atypical agent has been shown to be superior to placebo in several randomized, double-blind comparisons in the treatment of acutely manic patients. Olanzapine at a dose of 5–20 mg/day resulted in substantially greater improvement as compared to placebo-treated subjects in a 3-week study (Tohen et al., 1999). The olanzapine treated patients response rate (defined as a reduction in YMRS score) was 48%, compared with 24% in the placebo-treated group. The difference favoring olanzapine was significant after the third week of the study. A second randomized, placebo-controlled study yielded similar results, except for the difference in efficacy between olanzapine and placebo which appeared after only 1 week of treatment and was sustained throughout the duration of the trial (Tohen et al., 2000). In a 3-week randomized, double-blind study comparing olanzapine (5–20 mg/day, mean dose 17.4 mg/day) with divalproex (500–2500 mg/day, mean dose 1 401 mg/day, mean blood level of 82 mg/ml) for the treatment of acute mania (Tohen et al., 2002), a slight but significant advantage was observed for olanzapine over divalproex. Clinical improvement was evaluated using the percentage of patients achieving a greater than 50% decrease in YMRS scores, remission was evaluated using the percentage of patients with a YMRS score of 12 or lower. A 44-week double-blind extension of this study found that after only 15 weeks of treatment, the efficacy of valproate was similar to that of olanzapine (Zajecka et al., 2002; Tohen et al., 2003). By contrast, Zajecka has reported similar efficacies for olanzapine and valproate in a 12-week randomized, double-blind study involving 120 patients with acute mania. As compared with the Lilly funded Tohen’ study, the Abbott-founded Zajeka’ study has used a lower dose of olanzapine (mean dose: 14.7 mg/day) and a higher dose of valproate (mean dose: 2115 mg/day compared to 1401 in the previous study) which might explained the different results observed between the two studies. In both studies, patients receiving olanzapine experienced significantly more side effects, especially somnolence and weight gain, than those in the valproate group. Compared with lithium, olanzapine has shown a greater efficacy as compared to lithium in a 4-week trial but olanzapine was associated with more frequent side effects which may limit its use (Niufan et al., 2008). A large pan-European naturalistic mania study has confirmed the efficacy of olanzapine as monotherapy or in combination with other medications in a broad spectrum of manic patients (Vieta et al., 2008). The most worrisome adverse effects of olanzapine are metabolic.

#### Quetiapine

Several case reports (Dunayevich and Strakowski, 2000), and retrospective case series have suggested a useful role for the atypical antipsychotic quetiapine when used as an adjunctive treatment for mania.

Two large international multicentre randomized, double-blind, placebo-controlled trials have evaluated the efficacy of quetiapine monotherapy (400–800 mg/day) in hospitalized manic patients. In the first one (Bowden et al., 2005), 302 patients were randomized to quetiapine (*n* = 1007), lithium (*n* = 98), or placebo (*n* = 95). At week 3, the decrease in YMRS scores was significantly greater for both drugs compared with placebo (*P* < 0,001); the extent of the improvement was virtually identical for the two drugs. The other study McIntyre et al. (2005) was of the same size using the same design, except for the active comparator which was haloperidol; the results obtained were similar, except for the effect of haloperidol at week 3 which was slightly more robust than that of quetiapine. In both studies, quetiapine has shown significantly greater efficacy than placebo.

Two large randomized, placebo-controlled trials (Sachs et al., 2004; Yatham et al., 2004) have examined the efficacy of adjunctive quetiapine in 402 hospitalized manic patients who were still substantially symptomatic (YMRS scores ≥20) after a minimum of 7 days of lithium or divalproex treatment. In both studies, the adjunction of quetiapine was associated with a significantly greater improvement as compared to mood stabilizer alone.

Regarding antipsychotics, it is important to note that patients were included in these adjunctive studies after at best a partial response to at least 1 week of previous treatment with mood stabilizers alone; thus the generalization of these results is limited.

The drop-out rates observed in these controlled studies due to side effects were comparable to placebo drop-outs.

#### Risperidone

In open studies conducted in manic patients, Tohen et al. (1996) have reported a 50% or greater reduction in manic symptoms in 10 out of 12 patients when risperidone was added to lithium. Keck et al. (1995), have studied a mixed group of patients receiving risperidone, noting that all nine bipolar patients with mania showed moderate to marked improvement when risperidone was added to a mood-stabilizing regimen consisting of lithium, valproate, or carbamazepine. Ghaemi and Sachs (1997) have found that 9 of 14 bipolar patients, most with mania or mixed states, were significantly improved by the addition of small doses of risperidone (<3 mg/day) to mood stabilizers.

A randomized, double-blind study of risperidone in combination with lithium, carbamazepine, or valproate showed a significantly faster decline in YMRS scores in patients receiving risperidone in addition to a mood stabilizer. The mean risperidone dose in this study was 4 mg/day (Yatham et al., 2003). In another prospective double-blind, placebo-controlled trial of risperidone as an adjunctive treatment for mania (to lithium or valproate, when the response was not adequate to previous treatment with these mood stabilizers), Sachs et al. (2002), have observed a greater improvement in YMRS scores with risperidone as compared to placebo at the end of the first, second, and third weeks respectively. Similar findings were reported by Yatham et al. (2004), who randomly added risperidone (*n* = 75) or placebo (*n* = 75) to lithium or valproate, with a significantly greater reduction in YMRS scores when combined treatment was used.

A 3-week-randomized study was conducted by Hirschfeld et al. (1999) in 134 manic patients receiving risperidone (mean dose of 4.1 mg/day) as compared to placebo (15 patients). The YMRS scores were significantly decreased in the risperidone group as early as the third day: 43% of those randomized to risperidone met the response criteria at endpoint versus only 24% in the placebo group. Remission rates (reduction of YMRS scores ≤12) were respectively of 38% for risperidone versus 24% for placebo.

In a randomized, double-blind trial of risperidone monotherapy, 45 manic patients were receiving risperidone (6 mg/day), haloperidol (10 mg/day), or lithium (800–1 200 mg/day) during a 3-week trial (Tohen et al., 2003). There were no differences in treatment outcomes among the three groups with a mean improvement of about 50–60% on the YMRS, as well as substantial improvement on general psychopathology and functioning scales (Segal et al., 1998).

Finally, in a recent randomized double-blind comparison study, risperidone and olanzapine has shown similar antimanic efficacy (Perlis et al., 2006). Overdosing of risperidone (>6 mg/day) should be avoided as this clearly impacts effectiveness due to EPSs and prolactin elevation (Grunze et al., 2009).

#### Ziprasidone

Ziprasidone monotherapy was tested for antimanic efficacy in three double-blind placebo-controlled studies which confirmed its antimanic efficacy. Despite of its good metabolic profile, due to the fear of potential cardiac toxicity, ziprasidone is not available in some countries and its use is restricted in others (Grunze et al., 2009).

The antimanic efficacy of clozapine, zotepine, and paliperidone (a risperidone metabolite) needs to be confirmed.

Intramuscular formulations of aripiprazole and olanzapine have shown efficacy in agitated patients suffering from acute mania.

## Conclusion

All the current guidelines agree on first treating manic/hypomanic or mixed episodes with lithium, valproate, or some atypical antipsychotics as monotherapy, in order to minimize side effects. Clinical studies suggest that carbamazepine is weaker than lithium and that sodium valproate is superior to lithium in reducing symptoms of mania. Sodium valproate is used in patients resistant to lithium treatment. Among the atypical antipsychotics, olanzapine, and risperidone have been most extensively studied. In our opinion, atypical antipsychotics are very important in the treatment of mania because their sedative effects may be desirable in the short term treatment of mania. Yet aripiprazole at 30 mg alone showed efficacy even though it is not so sedating.

Combinations (atypical antipsychotics plus lithium or valproate) should be reserved for severe mania as first-line choice or as a second-line choice in mild or moderate mania in which a first-line medication has failed. In a recent meta-analysis of randomized controlled studies comparing one active antimanic drug with another one or with placebo including combination and augmentation studies, risperidone, olanzapine, and haloperidol have shown the best efficacy profile in the treatment of acute mania while lamotrigine, topiramate, and gabapentin were not significantly better than placebo (Cipriani et al., 2011). In another meta-analysis of co-therapy and monotherapy in acute bipolar mania, Smith et al. (2007) analyzed 8 RCTs including 1124 patients and found that significant reductions in mania (>50% reduction from baseline at YMRS scores) were shown for haloperidol, olanzapine, risperidone, and quetiapine as co-therapy combined with a mood stabilizer even if combination was less well tolerated as compared with monotherapy. Goodwin et al. (2009), during the ECNP consensus meeting, suggested that conventional two-arm trial designs could benefit form adding a third antipsychotic monotherapy arm in order to improve data from studies which compare and quantify the efficacy of antipsychotics in monotherapy or in combination.

In reality, less than 10% of acutely manic patients receive monotherapy, the number of medications in manic patients is at least two (Haro et al., 2010). Licht et al. (1997) has estimated that less than 20% of a screened naturalistic patient cohort fulfils all inclusion criteria for entering a randomized controlled trial (Licht et al., 1997).

In the case of carbamazepine treatment, no combination with other agents is recommended because of potential interactions. The recommendations suggest to change the ongoing carbamazepine treatment with another first-line treatment.

All guidelines agree on stopping the ongoing antidepressant medication during a manic/mixed episode (Nivoli et al., 2011). Despite this clear recommandation, clinicians continue to prescribe antidepressant medications in at least 15% of the manic and mixed patients (Rosa et al., 2010).

Most guidelines recommend continuation of treatment for 6–12 months after remission from acute mania however controlled studies are lacking except for lithium, olanzapine, and aripiprazole but data supporting this recommendation for these latter compounds are only grade B (WFSBP guidelines, Grunze et al., 2009). Which drug should be discontinued first in case of combination therapy or how doses may be reduced after remission remains based on clinical experience. In addition and despite the lack of strong data, some guidelines recommend that all patients should be offered maintenance treatment after an acute episode, the WFSBP recommends to consider the overall efficacy and tolerability of the medications in long term treatment in selection of a drug or a regimen for acute treatment of mania.

The question of which is the best strategy to face partial response is unanswered by the current guidelines. Evidence is lacking about when to stop a medication if the first choice treatment is inefficacious or leads to a partial response or when to add another medication (e.g., an atypical antipsychotic to the mood stabilizer). In addition, usually a 50% reduction in the YMRS or MRS is used as a response criterion but clear and valid definitions and assessments of partial response to treatment are needed.

In clinical practice, severity of mania and speed of onset of action are the primary arguments in first choice treatment of an acute manic episode.

In Europe, haloperidol remains a gold standard even if this drug is not registered in the treatment of acute mania. Lithium would be the ideal comparator except for the cases where mania is recurring in patients already receiving lithium for maintenance treatment with adequate dosage.

Direct comparative trials between atypical antipsychotics are still limited except for the comparison between olanzapine and risperidone. Others are either inconclusive, or not powered for comparison except for three studies showing that haloperidol is more powerful in the short term treatment of acute mania than olanzapine, quetiapine (Grunze et al., 2009).

Finally, so far, no psychological intervention has shown efficacy in controlled studies in comparison to a “placebo” intervention in mania (Gutierrez and Scott, 2004).

Rational therapeutic development in bipolar is hampered by a lack of pathophysiological model. However, there is a wealth of converging data on the role of dopamine in bipolar disorder. Pharmacological models suggest a role of increased dopaminergic drive in mania and the converse in depression. In Parkinson’s disease, administration of high-dose dopamine precursors can produce a “maniform” picture, which switches into a depressive analog on withdrawal. It is possible that in bipolar disorder there is a cyclical process, where increased dopaminergic transmission in mania leads to a secondary down regulation of dopaminergic receptor sensitivity over time. This may lead to a period of decreased dopaminergic transmission, corresponding with the depressive phase, and the repetition of the cycle. This model, if verified, may have implications for rational drug development (Berk et al., 2007).

## Conflict of Interest Statement

The authors declare that the research was conducted in the absence of any commercial or financial relationships that could be construed as a potential conflict of interest.
